# Prediction of herpes virus infections after solid organ transplantation: a prospective study of immune function

**DOI:** 10.3389/fimmu.2023.1183703

**Published:** 2023-07-03

**Authors:** Dina Leth Møller, Søren Schwartz Sørensen, Omid Rezahosseini, Daniel Bräuner Rasmussen, Nicoline Stender Arentoft, Josefine Amalie Loft, Michael Perch, Finn Gustafsson, Jens Lundgren, Thomas Scheike, Jenny Dahl Knudsen, Sisse Rye Ostrowski, Allan Rasmussen, Susanne Dam Nielsen

**Affiliations:** ^1^ Viro-immunology Research Unit, Department of Infectious Diseases 8632, Rigshospitalet, University of Copenhagen, Copenhagen, Denmark; ^2^ Department of Nephrology, Rigshospitalet, University of Copenhagen, Copenhagen, Denmark; ^3^ Department of Clinical Medicine, University of Copenhagen, Copenhagen, Denmark; ^4^ Department of Cardiology, Section for Lung Transplantation, Rigshospitalet, University of Copenhagen, Copenhagen, Denmark; ^5^ Department of Cardiology, Rigshospitalet, University of Copenhagen, Copenhagen, Denmark; ^6^ Centre of Excellence for Health, Immunity, and Infections, Rigshospitalet, University of Copenhagen, Copenhagen, Denmark; ^7^ Department of Biostatistics, University of Copenhagen, Copenhagen, Denmark; ^8^ Department of Clinical Microbiology, Rigshospitalet, University of Copenhagen, Copenhagen, Denmark; ^9^ Department of Clinical Immunology, Rigshospitalet, University of Copenhagen, Copenhagen, Denmark; ^10^ Department of Surgical Gastroenterology and Transplantation, Rigshospitalet, University of Copenhagen, Copenhagen, Denmark

**Keywords:** solid organ transplantation, herpes virus, cytomegalovirus, TruCulture®, immune functional assay, prediction

## Abstract

**Introduction:**

Herpes virus infections are a major concern after solid organ transplantation and linked to the immune function of the recipient. We aimed to determine the incidence of positive herpes virus (cytomegalovirus (CMV), Epstein-Barr virus (EBV), herpes simplex virus type 1/2 (HSV-1/2), and varicella zoster virus (VZV)) PCR tests during the first year post-transplantation and assess whether a model including immune function pre-transplantation and three months post-transplantation could predict a subsequent positive herpes virus PCR.

**Methods:**

All participants were preemptively screened for CMV, and EBV IgG-negative participants were screened for EBV during the first year post-transplantation. Herpes virus PCR tests for all included herpes viruses (CMV, EBV, HSV-1/2, and VZV) were retrieved from the Danish Microbiology database containing nationwide PCR results from both hospitals and outpatient clinics. Immune function was assessed by whole blood stimulation with A) LPS, B) R848, C) Poly I:C, and D) a blank control. Cytokine concentrations (TNF-α, IL-1β, IL-6, IL-8, IL-10, IL-12p40, IL-17A, IFN-α, and IFN-γ) were measured using Luminex.

**Results:**

We included 123 liver (54%), kidney (26%), and lung (20%) transplant recipients. The cumulative incidence of positive herpes virus PCR tests was 36.6% (95% CI: 28.1-45.1) during the first year post-transplantation. The final prediction model included recipient age, type of transplantation, CMV serostatus, and change in Poly I:C-induced IL-12p40 from pre-transplantation to three months post-transplantation. The prediction model had an AUC of 77% (95% CI: 61-92). Risk scores were extracted from the prediction model, and the participants were divided into three risk groups. Participants with a risk score <5 (28% of the cohort), 5-10 (45% of the cohort), and >10 (27% of the cohort) had a cumulative incidence of having a positive herpes virus PCR test at 5.8%, 25%, and 73%, respectively (p < 0.001)

**Conclusion:**

In conclusion, the incidence of positive herpes virus PCR tests was high, and a risk model including immune function allowed the prediction of positive herpes virus PCR and may be used to identify recipients at higher risk.

## 1 Introduction

Herpes virus infections are a major concern after solid organ transplantation (SOT) (1, 2) and may cause direct morbidity and mortality as well as indirect consequences, including increased rates of other infections and graft loss (1, 3–7). Immunosuppressive medication post-transplantation increases the risk of herpes virus infection significantly (3). Therefore, preventive measures are commonly used, including preemptive screening and antiviral prophylaxis during the early post-transplantation phase (3).

The herpes virus family consists of eight human herpes viruses, of which cytomegalovirus (CMV), Epstein-Barr virus (EBV), herpes simplex virus type 1/2 (HSV-1/2), and varicella zoster virus (VZV) have significant clinical impact post-transplantation (1, 6). Herpes viruses are DNA viruses with the ability to form latent infections that may reactivate in immunosuppressed individuals (8). The prevalence of seropositivity is high in adults, with 60-90% being seropositive for CMV (8), 90-95% for EBV (4), 59.7% for HSV-1 (9), 21.2% for HSV-2 (9), and >90% for VZV (6), resulting in a large SOT population at risk of reactivation. Furthermore, in seronegative individuals transplanted with organs from seropositive donors, herpes virus infections can be donor-derived, causing a primary infection in an unprotected and immunosuppressed recipient (4).

The risk of herpes virus reactivation is linked to the immune function of the recipient, and other known risk factors are serostatus, older age, leucopenia, and particular immunosuppressive drugs or regimes (6, 10–12). However, no standardized method of assessing the recipients’ overall immune function is currently used in clinical practice. Previous studies have investigated CMV antigen interferon-γ (IFN-γ) releasing assays to guide CMV prophylaxis with promising results (13, 14). However, these studies only assessed CMV-specific immunity. Interestingly, a new commercially available and highly standardized method (TruCulture®) to asses overall immune function has been investigated in other settings and may be used in SOT recipients (15–17). We hypothesized that individual immune function contributes to the observed differences in the risk of herpes virus infections. Thus, creating a prediction model combining known risk factors with an assessment of the immune function by TruCulture® might improve our ability to identify recipients at risk of herpes virus infection post-transplantation.

In a cohort of SOT recipients, we aimed to determine the incidence of positive herpes virus polymerase chain reaction (PCR) tests during the first year post-transplantation. Furthermore, we assessed and compared immune function in recipients with and without a positive herpes virus PCR test by TruCulture® and investigated whether a model including a functional immune assay could be used to predict positive herpes virus PCR tests.

## 2 Materials and methods

The study is a prospective cohort study including adult (≥ 18 years) single-organ SOT (liver, lung, or living-donor kidney transplantation) recipients transplanted at Rigshospitalet, Copenhagen University Hospital, Copenhagen, Denmark from March 1^st^, 2018 to January 31^st^, 2021.

We included SOT recipients with both a pre-transplantation and three months post-transplantation TruCulture® analysis. This entails that all study participants survived the first three months post-transplantation.

Herpes virus PCR tests were retrieved through the Centre of Excellence for Personalized Medicine of Infectious Complications in Immune Deficiency (PERSIMUNE) data repository (18) which contains information from national registries and clinical databases, including the Danish Microbiology Database (MiBa) (19). MiBa contains nationwide herpes virus PCR results from both general practice, hospitals, and outpatient clinics.

Clinical characteristics were collected from patient’s records, including information on rejection episodes, immunosuppressive medication, and antiviral prophylaxis. In addition, recipient and donor serostatus for CMV, EBV, HSV-1/2, and VZV prior to transplantation was collected from the Management of Post-transplant Infections in Collaborating Hospitals (MATCH) program. The MATCH program is a personalized screening program for viral infections post-transplantation at our transplantation center, based on donor/recipient serostatus for common viruses (20, 21). Information about vaccination against herpes zoster was collected from the nationwide Danish Vaccination Register (DDV), which has been mandatory since 2015 (22). Leucocyte and lymphocyte counts were collected on the day closest to the day of the three months post-transplantation sample (± 14 days).

The study was conducted in accordance with the Helsinki Declaration. Written informed consent was obtained from all participants in the study. The study was approved by the Health Authorities (3-3013-1060/1), the Regional Committee on Health Research Ethics (H-17024315), and the Data Protection Agency (RH-2016-47, RH-2015-04, I-Suite 03605). The study has been registered at clinicaltrials.gov (NCT03847285).

### 2.1 Herpes virus screening

The study outcome was PCR tests positive for the following herpes viruses: CMV, EBV, HSV-1/2, and VZV. All participants were preemptively screened for CMV in intervals depending on their serostatus, with a minimum of six CMV PCR tests during the first year post-transplantation (20). Additionally, EBV IgG-negative participants were screened for EBV during the first year post-transplantation (23). There was no systematic screening for HSV-1/2 or VZV. In addition, PCR tests were performed for all herpes viruses on clinical suspicion. A positive PCR test was defined as a PCR test above the lower limit of detection regardless of the assay used from any site in Denmark.

### 2.2 Herpes virus prophylaxis

All participants received herpes virus chemoprophylaxis with either valganciclovir or valaciclovir post-transplantation depending on their CMV serostatus. In the liver transplant recipients, recipients with CMV serostatus Donor positive/Recipient negative (D+/R-) and D+/R+ received three months of valganciclovir, whereas recipients with CMV serostatus D-/R- and D-/R+ received three months of valaciclovir for the prevention of HSV-1/2 and VZV. In the lung transplant recipients, recipients with CMV serostatus D+/R-, D+/R+, and D-/R+ received three months of valganciclovir, whereas seronegative recipients with seronegative donors (D-/R-) received three months of valaciclovir for the prevention of HSV-1/2 and VZV infections. Ten lung transplanted participants (42%) received valganciclovir for six to 12 months as part of a clinical trial. All kidney transplant recipients received three months of valganciclovir.

### 2.3 Immunosuppressive treatment and rejections

Induction therapy was used as standard treatment in the kidney and lung transplanted recipients who received basiliximab and anti-thymocyte globulin (ATG), respectively, and in selected liver transplant recipients (**Table 1**). The immunosuppressive maintenance treatment consisted of tacrolimus, mycophenolate mofetil (MMF), and glucocorticoids for the liver and kidney transplanted recipients and ciclosporin, MMF, and glucocorticoids for the lung transplanted recipients.

**Table 1:** Characteristics of the cohort.

	All participants	Liver transplanted participants	Kidney transplanted participants	Lung transplanted participants	P-values*
	n = 123	n = 67 (54%)	n = 32 (26%)	n = 24 (20%)	
Age at Tx, median (IQR)	50.5 (38–57)	50.1 (37–57)	45.2 (38–53)	55.1 (51–60)	0.04
Males, n (%)	75 (61%)	41 (61%)	21 (66%)	13 (54%)	0.75
Retransplantation	4 (3%)	0 (0%)	4 (13%)	0 (0%)	0.005
Immunosuppressive treatment					
Induction therapy					
No induction	47 (38%)	47 (70%)	0 (0%)	0 (0%)	<0.001
Basiliximab	48 (39%)	18 (27%)	30 (94%)	0 (0%)	
ATG	25 (20%)	0 (0%)	1 (3%)	24 (100%)	
Other^a^	3 (3%)	2 (3%)	1 (3%)	0 (0%)	
Maintenance immunosuppressive treatment in the first three months post-transplantation^b^					
Calcineurin inhibitors					
Tacrolimus	100 (81%)^b^	61 (91%)	32 (100%)^b^	7 (29%)	<0.001
Ciclosporin	24 (20%)^b^	6 (9%)	1 (3%)^b^	17 (71%)	
mTor inhibitors	4 (3%)^b^	2 (3%)^b^	2 (6%)^b^	0 (0%)	0.518
Antiproliferatives					
Mycophenolate	122 (99%)^b^	66 (99%)^d^	32 (100%)^b^	24 (100%)^b^	0.83
Azathioprine	5 (4%)^b^	2 (3%)^d^	2 (6%)^b^	1 (4%)^b^	
Glucocorticoids	123 (100%)^c^	67 (100%)^c^	32 (100%)	24 (100%)	0.999
Acute rejections during the first year post-transplantation treated with high-dose methylprednisolone					
Participants with acute rejection	40 (33%)	12 (18%)	18 (56%)	10 (42%)	<0.001
After the three months blood sample	12 (10%)	4 (6%)	3 (9%)	5 (21%)	0.109
Acute rejections, no.	45 (100%)	12 (27%)	20 (44%)	13 (29%)	0.075
Acute rejections treated with^d^					
High-dose methylprednisolone	45 (100%)^d^	12 (100%)^d^	20 (100%)^d^	13 (100%)^d^	0.999
ATG^e^	3 (7%)^d^	1 (8%)^d^	2 (10%)^d^	0 (0%)	
Apheresis^e^	3 (7%)^d^	0 (0%)	2 (10%)^d^	1 (8%)^d^	

*P-values for differences between the three types of transplantation.

a: Ciclosporin A and daclizumab.

b: Add to more than 100% since some participants changed treatment during the period.

c: In one participant, the glucocorticoid treatment was stopped before three months post-transplantation due to severe side effects.

d: Add to more than 100% since some rejections were treated with several approaches.

e: All previously treated with high-dose methylprednisolone.

Rejection episodes were defined as either biopsy-confirmed or suspected rejections treated with high-dose methylprednisolone alone or in combination with other treatments.

### 2.4 TruCulture® assay

The TruCulture® assay has been described previously (15, 16, 24). In brief, 1 mL of whole blood was transferred into four TruCulture® tubes (Myriad RBM, Autin, TX, USA) within one hour of sampling. The tubes contained either A) Bacterial endotoxin (lipopolysaccharide, LPS) from E. Coli O111:B4 (cat.#782-001261), B) Resiquimod R848 (R848) (cat.#782-001264), C) Polyinosinic:polycytidylic acid (Poly I:C) (cat.#782-001282), and D) a blank control containing only TruCulture® media (cat.#782-001086). After 22 hours (+/-30 min) of incubation at 37°C, the supernatant was harvested according to the manufacturer’s instructions. The supernatants were stored at -80°C until use.

Cytokine concentrations (TNF-α, IL-1β, IL-6, IL-8, IL-10, IL-12p40, IL-17A, IFN-α, and IFN-γ) were measured in singlets using a 9-plex Luminex assay (R&D Systems, BIO-Techne LTD, Minneapolis, MN, USA) on a Luminex 200 instrument (R&D Systems) according to the manufacturer’s recommendations. All cytokine concentrations were measured in pg/mL.

The TruCulture® assay was designed to investigate immunological signaling pathways through toll-like receptor (TLR) stimulation. LPS stimulates the extracellular TLR-4 mimicking infection with a Gram-negative bacteria, whereas R848 and Poly I:C stimulate the intracellular TLR-7/TLR-8 and TLR-3, respectively, thereby mimicking viral infections.

### 2.5 Statistics

Continuous data were presented as medians with interquartile ranges (IQR) and categorical data as proportions. Differences in continuous and categorical data were calculated using the Mann-Whitney U test or Kruskal-Wallis test and the Chi^2^ test or Fisher’s exact test, respectively. The cumulative incidence of positive herpes virus PCR tests was calculated using the Aalen-Johansen estimator with re-transplantation and death as competing risks (R-package *prodlim*). Differences in cumulative incidence were compared using Grays test (R-package *cpmrsk*). To assess important covariates for the prediction model, cumulative incidences stratified by either age, sex, type of transplantation, and CMV serostatus were calculated. Furthermore, as sensitivity analyses cumulative incidences stratified by the type of antiviral chemoprophylaxis (valganciclovir vs. valaciclovir), type of induction therapy (ATG vs. basiliximab vs. other vs. no induction therapy), and type of calcineurin inhibitor used during used the first 3 months (tacrolimus vs. ciclosporin) were calculated.

Differences in induced cytokine concentrations or changes in cytokine concentrations in participants with or without herpes infection were compared using exact Mann-Whitney U test for non-parametric unpaired continuous data (R-package *coin*). To account for multiple comparisons, p-values were corrected by the Benjamini-Hochberg method (R-package *stats*). To accommodate cytokine concentrations below the measuring point, which transcripted to 0 pg/mL; 1% of the lowest measured cytokine concentration was added to all cytokine concentrations in the logarithmic illustrations of the cytokine concentrations. Sensitivity analyses were conducted in participants with CMV serostatus R+ and D+/R-. Furthermore, sensitivity analyses of only positive CMV PCR tests were conducted in all participants and participants with CMV serostatus R+ and D+/R-.

For the prediction of positive herpes virus PCR tests from three to 12 months post-transplantation, all cytokines at three months and the change between pre-transplantation and three months post-transplantation were screened using Cox proportional hazard regression models (R-package *survival*). All participants were followed from their second blood sample at three months post-transplantation to death, re-transplantation, or one year post-transplantation, whichever came first. The models were adjusted for age, type of transplantation, and CMV serostatus.

The dataset was subsequently split into a testing cohort (2/3) and a validation cohort (1/3), where the testing cohort served to make the model parameters and the validation cohort served to validate the model in a different group of participants. The significant cytokine from the screening ability to predict positive herpes virus PCR tests was assessed using Brier scores (R-package *riskRegression*). For validation purposes, the splitting was done 10 times, and the average Brier scores were used. Models including IFN-α were assessed separately since only a subgroup of 86 participants (70%) had IFN-α measurements. For the model with the best Brier score, a receiver operating characteristics (ROC) curve with area under the curve (AUC) was drawn (R-package *riskRegression*). Finally, a risk score was constructed using the linear predictors from the best performing model using the entire dataset (**Supplementary Figure S1**). Sensitivity, specificity, positive predictive values (PPV), and negative predictive values (NPV) were calculated for ten different cut-off values of the risk score (R-package *epiR*).

The best performing models prediction ability was further investigated in a sensitivity analysis by adding the leucocyte or lymphocyte count of the participants. Furthermore, sensitivity analyses were conducted in participants with CMV serostatus R+. Lastly, sensitivity analyses, including only positive CMV PCR tests as the outcome, were conducted.

P-values < 0.05 were considered statistically significant. All statistical analyses were conducted in the software R version 3.6.1 (R Foundation for Statistical Computing, Vienna, Austria).

## 3 Results

### 3.1 Characteristics of the study participants

In this study, 123 SOT recipients were included consisting of 67 liver (54%), 32 kidney (26%), and 24 lung (20%) transplant recipients. Clinical characteristics are presented in **Table 1**. A total of 40 participants (33%) had allograft rejection treated with high-dose methylprednisolone during the first year post-transplantation at a median of 13 days (IQR: 8-88 days) post-transplantation (**Table 1**).

Serostatus for herpes viruses was collected prior to transplantation. For CMV, 19% had a high-risk serotype (D+/R-), 70% had an intermediate-risk serotype (D+/R+ and D-/R+), and 11% had a low-risk serotype (D-/R-) (**Table 2**). Serostatus for EBV, HSV-1/2, and VZV are shown in **Table 2**. Most of the participants received valganciclovir as chemoprophylaxis (83%) for a median of 94 days (IQR 90-109) (**Table 2**).

**Table 2:** Serostatus at transplantation and antiviral prophylaxis.

Serostatus at transplantation					
	All participants n = 123	Liver transplanted participants n = 67 (54%)	Kidney transplanted participants n = 32 (26%)	Lung transplanted participants n = 24 (20%)	p-values*
CMV					
D+/R+ D+/R- D-/R+ D-/R-	63 (51%) 23 (19%) 23 (19%) 14 (11%)	36 (54%) 11 (16%) 12 (18%) 8 (12%)	15 (46%) 5 (16%) 8 (25%) 4 (13%)	12 (50%) 7 (29%) 3 (13%) 2 (8%)	0.756
*Unknown for either donor or recipient*	0 (0%)	0 (0%)	0 (0%)	0 (0%)	
EBV					
D+/R+ D+/R- D-/R+ D-/R-	95 (77%) 8 (7%) 3 (2%) 0 (0%)	47 (70%) 5 (7%) 1 (2%) 0 (0%)	28 (88%) 3 (9%) 1 (3%) 0 (0%)	20 (83%) 0 (0%) 1 (4%) 0 (0%)	0.554
*Unknown for either donor or recipient* Unknown/R+ Unknown/R- Unknown	17 (14%) 16 (13%) 0 (0%) 1 (1%)	14 (21%) 14 (21%) 0 (0%) 0 (0%)	0 (0%) 0 (0%) 0 (0%) 0 (0%)	3 (13%) 2 (8%) 0 (0%) 1 (4%)	
HSV-1/2					
D+/R+ D+/R- D-/R+ D-/R-	49 (40%) 18 (15%) 18 (15%) 6 (4%)	24 (36%) 6 (9%) 10 (15%) 2 (3%)	15 (47%) 10 (31%) 2 (6%) 4 (13%)	10 (42%) 2 (8%) 6 (25%) 0 (0%)	0.063
*Unknown for either donor or recipient* Unknown/R+ Unknown/R- Unknown	32 (26%) 23 (19%) 6 (5%) 3 (2%)	25 (37%) 17 (25%) 5 (7%) 3 (4%)	1 (3%) 1 (3%) 0 (0%) 0 (0%)	6 (25%) 5 (21%) 1 (4%) 0 (0%)	
VZV					
D+/R+ D+/R- D-/R+ D-/R-	86 (70%) 0 (0%) 5 (4%) 0 (0%)	41 (61%) 0 (0%) 2 (3%) 0 (0%)	29 (91%) 0 (0%) 2 (6%) 0 (0%)	16 (67%) 0 (0%) 1 (4%) 0 (0%)	0.999
*Unknown for either donor or recipient* Unknown/R+ Unknown/R- Unknown	32 (26%) 31 (25%) 0 (0%) 1 (1%)	24 (36%) 24 (36%) 0 (0%) 0 (0%)	1 (3%) 1 (3%) 0 (0%) 0 (0%)	7 (29%) 6 (25%) 0 (0%) 1 (4%)	
Vaccination against herpes Zoster (All Shingrix vaccines) Prior to transplantation Within the first year post-transplantation^a^	16 (13%) 2 (2%) 14 (11%)	14 (21%) 2 (3%) 12 (18%)	0 (0%) 0 (0%) 0 (0%)	2 (8%) 0 (0%) 2 (8%)	0.006 0.999 0.015
Antiviral prophylaxis					
Type of prophylaxis Valganciclovir Valaciclovir	102 (83%) 21 (17%)	48 (72%) 19 (28%)	32 (100%) 0 (0%)	22 (92%) 2 (8%)	<0.001
Median length in days of prophylaxis post-transplantation (IQR)	94 (90–109)	96 (91–106)	92 (90–95)	95 (89–198)	0.188
No. of participants stopping prophylaxis before three months post-transplantation (+/- 14 days) *Missing data on the end of prophylaxis*	9 (7%)^b^ 1 (1%)	5 (7%) 1 (1%)	2 (6%) 0 (0%)	2 (8%) (0%)	0.999 0.999

*P-values for differences between the three types of transplantation.

a: Participants with at least one of the two vaccination shots after transplantation will be noted in the group “Within the first year post-transplantation”.

b: Reasons for an early end of CMV prophylaxis: 5 participants had leukopenia or beginning leukopenia, 1 participant had CMV infection and started high dose valganciclovir, 1 participant had decreased kidney function, 1 participant had increased liver enzymes, and 1 participant ended for unknown reasons.

During the follow-up period from three to 12 months post-transplantation, five participants died at a median of 274 days (IQR 223-277) post-transplantation. None of the study participants were retransplanted or graftectomized. One kidney transplant recipient returned to permanent dialysis but continued immunosuppressive medication and remained in the study.

### 3.2 Herpes virus PCR tests

All participants were tested for CMV during the first year post-transplantation, while only a subset was tested for EBV (71%), HSV-1/2 (21%), and VZV (20%) (**Table 3**). A total of 2633 herpes virus PCR tests were performed during the first year post-transplantation. Of these, 361 PCR tests were positive in 45 participants (37%). These included 301 positive CMV PCR tests in 39 (32%) participants, 41 positive EBV PCR tests in 10 (8%) participants, 15 positive HSV-1/2 PCR tests in 5 (4%) participants, and 4 positive VZV PCR tests in 3 (2%) participants.

**Table 3:** Herpes virus infections in the cohort.

	All participants	Liver transplanted participants	Kidney transplanted participants	Lung transplanted participants	p-values*
	n = 123	n = 67 (54%)	n = 32 (26%)	n = 24 (20%)	
No. of tested participants (%) for					
CMV	123 (100%)	67 (100%)	32 (100%)	24 (100%)	0.999
EBV	87 (71%)	43 (64%)	20 (63%)	24 (100%)	<0.001
HSV-1/2	26 (21%)	6 (9%)	2 (6%)	18 (75%)	<0.001
VZV	24 (20%)	2 (3%)	2 (6%)	18 (75%)	<0.001
Test					
Type of test, n (% of conducted tests)					
CMV	2099 (80%)	974 (84%)	423 (83%)	702 (73%)	<0.001
EBV	371 (14%)	168 (14%)	72 (14%)	131 (14%)	
HSV-1/2	106 (4%)	14 (1%)	12 (2%)	80 (8%)	
VZV	57 (2%)	7 (1%)	5 (1%)	45 (5%)	
Test material, n (% of conducted tests)					
Blood	2292 (87%)	1134 (98%)	501 (98%)	657 (68%)	<0.001
Airway	307 (11%)	16 (1%)	7 (1%)	284 (30%)	
Swab	18 (1%)	4 (<1%)	2 (<1%)	12 (1%)	
CSF	8 (<1%)	3 (<1%)	0 (0%)	5 (<1%)	
Other/Unknown^a^	8 (<1%)	6 (<1%)	2 (<1%)	0 (0%)	
Results of tests					
All herpes viruses, n (% of conducted tests)					
Positive	361(14%)	163 (14%)	67 (13%)	131(14%)	0.984
Negative	2251 (85%)	990 (85%)	441 (86%)	820 (85%)	
Inconclusive	21(1%)	10 (1%)	4 (1%)	7 (1%)	
Positive, n (no. participants)					
CMV	301 (39)	131 (19)	56 (6)	114 (14)	<0.001
EBV	41 (10)	30 (4)	5 (2)	6 (4)	
HSV-1/2	15 (5)	1 (1)	6 (1)	8 (3)	
VZV	4 (3)	1 (1)	0 (0)	3 (2)	
Cumulative incidence of herpes virus infection					
Cumulative incidence of herpes virus infection	36.6% (CI:28.1-45.1)	32.8% (CI: 21.6-44.1)	21.9% (CI:7.6-36.2)	66.7% (CI: 44.8-85.5)	<0.001
Cumulative incidence of					
CMV	31.7% (CI:23.5-39.9)	28.4% (CI: 17.6-39.2)	18.8% (CI: 5.2-32.3)	58.3% (CI: 38.6-78.1)	0.003
EBV	8.1% (CI: 3.3-1.3)	6.0% (CI: 0.3-11.6)	6.3% (CI: 0.0-14.6)	16.7% (CI: 1.8-31.6)	0.214
HSV-1/2	4.1% (CI: 0.6-7.6)	1.5% (CI: 0.0-4.4)	3.1% (CI: 0.0-9.2)	12.5% (CI: 0.0-25.7)	0.064
VZV	2.4% (CI: 0.0-5.2)	1.5% (CI: 0.0-4.4)	0.0% (CI: 0.0-0.0)	8.3% (CI: 0.0-19.4)	0.104

*P-values for differences between the three types of transplantation.

a: Other/unknown consists of Urine (n=2), Stool (n=1), Unknown (n=5).

**Figure 1:**
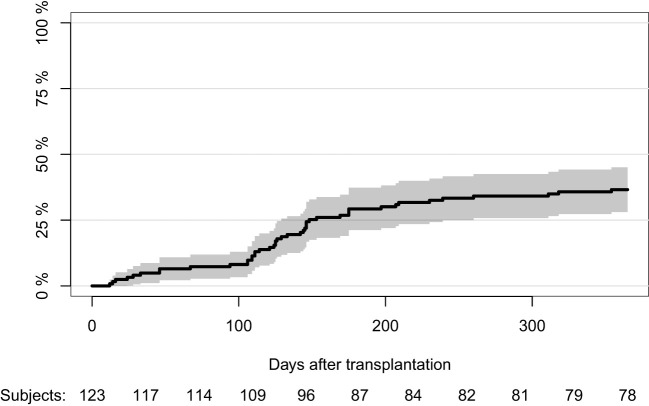
Cumulative incidence of first positive herpes virus PCR test during the first year post-transplantation.

The cumulative incidence of positive herpes virus PCR tests was 36.6% (CI:28.1-45.1) during the first year post-transplantation (**Figure 1**). Cumulative incidences of different herpes viruses are shown in **Table 3**. The cumulative incidence of positive herpes virus PCR tests was higher for lung transplant recipients than for liver and kidney transplant recipients (66.7% (CI: 44.8-85.5) vs. 32.8% (CI: 21.6-44.1) and 21.9% (CI: 7.6-36.2), p<0.001). Furthermore, age at transplantation (p=0.020), CMV serostatus (p<0.001), the type of induction therapy (p<0.001), and the type of calcineurin inhibitor used during the first three months (p<0.001) were associated with the cumulative incidence of positive herpes virus PCR tests, whereas the cumulative incidence was not affected by sex (p=0.228) or type of antiviral chemoprophylaxis (p=0.109) (**Supplementary Figures S2-5**).

### 3.3 Immune function in participants with and without positive herpes virus PCR tests

**Figure 2:**
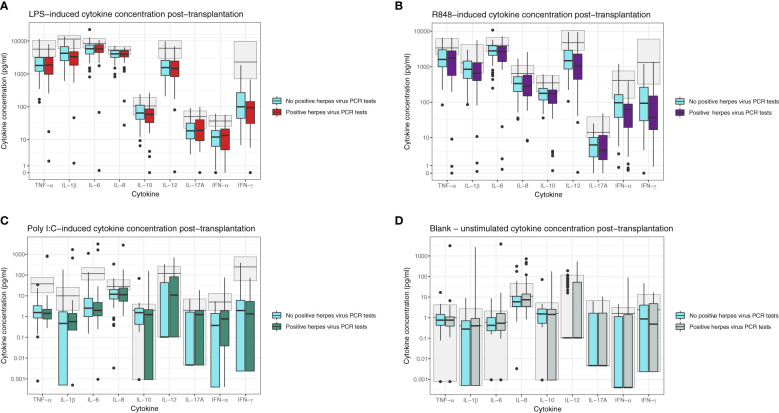
Induced cytokine concentration at three months post-transplantation in participants with (boxplots in red/purple/green/grey) or without (blue boxplots) subsequent positive herpes PCR tests for **(A)** LPS-induced cytokines, **(B)** R848-induced cytokines, **(C)** Poly I:C-induced cytokines, and **(D)** Unstimulated (Blank) cytokines. All cytokine concentrations are reported in pg/mL. The light grey boxplots in the background indicate the 5-95% reference interval based on healthy individuals. No p-values were significant after the Benjamini-Hochberg correction for multiple comparisons.

Forty-one participants (33%) had a positive herpes virus PCR test between the blood sample at three months and end of follow-up 12 months post-transplantation. Characteristics of the participants are shown in **Table 4**. These participants did not have significantly different induced cytokine concentrations at three months post-transplantation compared to participants without subsequent positive herpes PCR tests (**Figures 2A–C**). In the unstimulated samples (blank), IL-12p40 was increased in participants with later positive herpes virus PCR test (p=0.030). However, there was no significant difference after correction for multiple comparisons (**Figure 2D**). In a sensitivity analysis, participants with CMV serotype R+ (n = 86) or D+/R- (n = 23) were assessed separately. Participants with CMV serotype R+ and subsequent positive herpes virus PCR test had increased Poly I:C-induced IL-12p40, whereas D+/R- participants with subsequent positive herpes virus PCR test had increased Poly I:C-induced IL-17A (p=0.012 and p=0.025, respectively). However, no differences were significant after correction for multiple comparisons.

**Table 4:** Recipients with positive herpes virus PCR after the second TruCulture® sample.

	Recipients with later positive herpes virus PCR (n = 41)	Recipients without later positive herpes virus PCR (n= 82)	*p-values*
Median age (IQR) in years	53.6 (38.6-60.3)	49.8 (37.0-56.7)	0.07
Sex (% males)	35%	46%	0.3
Type of transplant			0.035
Liver	21 (51%)	46 (56%)	
Kidney	7 (17%)	25 (30%)	
Lung	13 (32%)	11 (14%)	
CMV serostatus prior to transplantation^a^			<0.001
D+/R+	19 (46%)	44 (54%)	
D+/R-	16 (39%)	7 (8%)	
D-/R+	6 (15%)	17 (21%)	
D-/R-	0 (0%)	14 (17%)	
Type of antiviral prophylaxis			0.045
Valganciclovir	38 (93%)	64 (78%)	
Valaciclovir	3 (7%)	18 (22%)	
Length of antiviral prophylaxis (IQR) in days	95 (90-116)	94 (90-105)	0.6
No. of recipients with rejection in the first three months post-transplantation	9 (22%)	22 (27%)	0.7
Median time to positive herpes PCR after TruCulture sample (IQR) in days	49 (24-84)	–
Type of first positive PCR			
CMV	34 (83%)	–	
EBV	3 (7%)		
HVS-1/2	2 (5%)		
VZV	2 (5%)		
No. of recipients positive for >1 type of herpes virus (% co-occurring)	9 (55%)	–	
Median positive PCR samples per recipient according to the type of herpes virus (IQR)			
CMV	6 (4-11)	–	
EBV	3 (1-4)		
HSV-1/2	3 (1-4)		
VZV	1 (1-2)		

a: No differences in EBV serostatus (p=0.400), HSV serostatus (p=0.700), or VZV serostatus (p=0.5).

**Figure 3:**
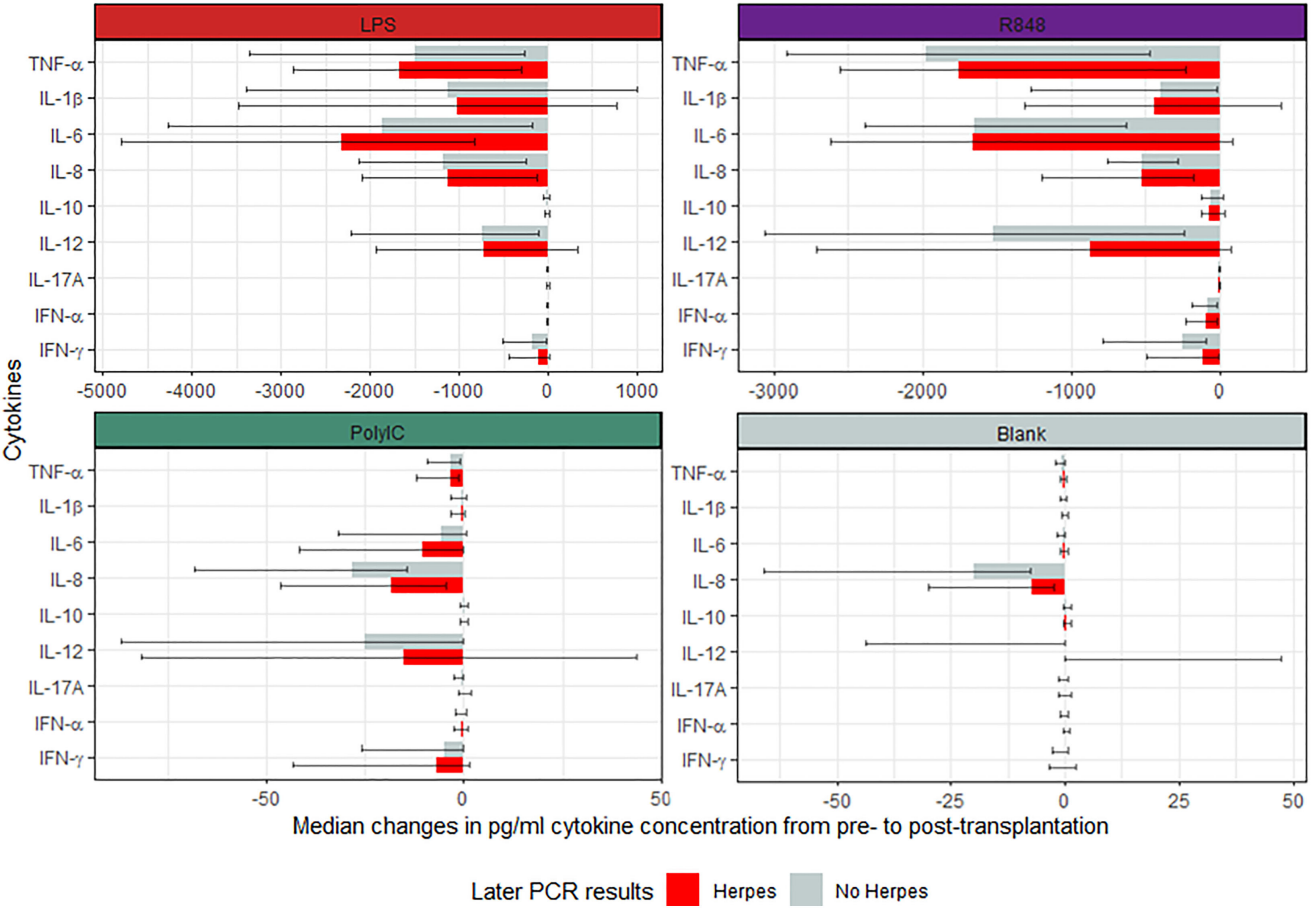
Median change in induced cytokine concentration pre- and post-transplantation in participants with or without subsequent positive herpes PCR tests. All cytokine concentrations are reported in pg/mL. No p-values were significant after the Benjamini-Hochberg correction for multiple comparisons.

Similarly, when comparing the changes in cytokine concentration from pre-transplantation to three months post-transplantation, there were no significant differences between participants with or without later positive herpes virus PCR test after correction for multiple comparisons (**Figure 3**). However, prior to the correction, Poly I:C-induced IL-8 and IL-17A, and unstimulated IL-8, IL-12p40, and TNF-α concentrations were higher in the group who subsequently presented with a positive herpes virus PCR test. In the sensitivity analysis of participants with CMV serotype R+, Poly I:C-induced IL-8 and unstimulated IL-8, IL-12p40, and TNF-α were increased in participants with subsequent positive herpes virus PCR tests. However, no differences were significant after correction for multiple comparisons. In addition, no differences were observed between D+/R- participants with or without subsequent positive herpes virus PCR tests.

There was a median of 45 days (IQR: 24-84) from the second blood sample to the positive PCR tests. However, five participants had a concurrent herpes virus infection during the blood sampling (defined as a positive herpes virus PCR test within seven days prior to blood sampling, including at the time of blood sampling). A sub-analysis without these participants was conducted, yielding the same results for both the cytokine concentrations at three months and the change in cytokine concentrations. Furthermore, three participants received antiviral chemoprophylaxis during the entire first year. A sensitivity analysis without these participants was conducted, yielding identical results after correction for multiple comparisons.

### 3.4 Prediction of positive herpes virus PCR tests post-transplantation

**Figure 4:**
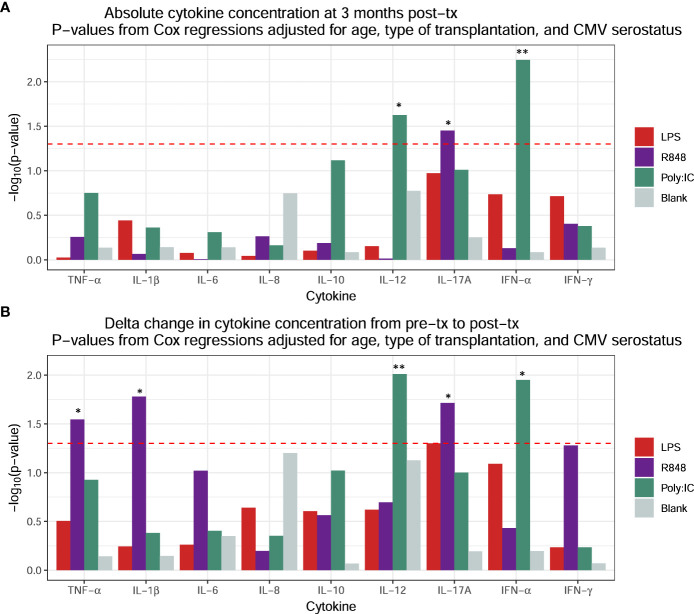
Screening for associations between subsequent positive herpes virus PCR tests and induced cytokine concentrations using Cox proportional hazards regressions adjusted for age, type of transplantation, and CMV serostatus for **(A)** absolute cytokine concentrations at three months post-transplantation and **(B)** changes in cytokine concentration from pre-transplantation to post-transplantation. P-values are represented as: * <0.05 and ** <0.01.

For our prediction model of positive herpes virus PCR test post-transplantation, all cytokine concentrations at three months and changes in cytokine concentrations from pre-transplantation to three months post-transplantation (abbreviated Δ) were screened for associations with positive herpes virus PCR tests using adjusted Cox proportional hazards regressions. These regressions were adjusted for age, type of transplantation, and CMV serostatus. This yielded a total of eight significant cytokine associations: High absolute concentrations at three months post-transplantation of Poly I:C-induced IFN-α and IL-12p40 and R848-induced IL-17A (**Figure 4A**) and increased positive changes (Δ) in Poly I:C-induced IFN-α and IL-12p40, and R8484-induced IL-17A, IL-1β, and TNF-α (**Figure 4B**).

Prediction models for positive herpes virus PCR tests, including the eight significantly associated cytokines one at a time combined with age, type of transplantation, and CMV serostatus, were tested against each other and evaluated using the Brier scores. The prediction models yielded average Brier scores ranging from 17.45-19.95 (**Table 5**). The best performing model was the model including Δ Poly I:C-induced IL-12p40, where higher Poly I:C-induced IL-12p40 concentration at 3 months compared to pre-transplantation (increased positive change in Δ Poly I:C-induced IL-12p40) was associated with positive herpes virus PCR tests.

**Table 5:** Brier and AUC scores.

Model	Average Brier Score (lower-upper score)	Average AUC Score (lower-upper score)
Age, type of transplantation, and CMV serostatus at transplantation	19.25 (12.65-25.83)	70 (52-88)
+ Poly I:C-induced IL-12 at three months	18.04 (11.67-24.42)	76 (59-91)
+ R848-induced IL-17A at three months	19.39 (12.8-25.97)	72 (55-89)
+ Change in Poly I:C-induced IL-12	17.45 (11.35-23.56)	77 (61-92)
+ Change in R848-induced IL-17A	19.22 (12.50-25.99)	73 (56-89)
+ Change in R848-induced IL-1β	19.95 (13.17-26.75)	72 (55-89)
+ Change in R848-induced TNF-α	19.66 (12.71-26.58)	72 (55-89)

When assessing the participants with complete IFN-α measurements, the model including absolute Poly I:C-induced IL-12p40 at three months performed best (**Supplementary Table S2**).

To further validate our model, a subanalysis was performed, excluding the five participants with concurrent herpes virus infection at time of blood sampling. This analysis equally found the model including Δ Poly I:C-induced IL-12p40 superior to the other models (**Supplementary Table S3**). Furthermore, a subanalysis was performed excluding the three participants who received antiviral chemoprophylaxis during the entire first year, where the model including Δ Poly I:C-induced IL-12p40 performed superior to the other models (**Supplementary Table S4**).

### 3.5 Evaluation of the prediction model for positive herpes virus PCR test post-transplantation

**Figure 5:**
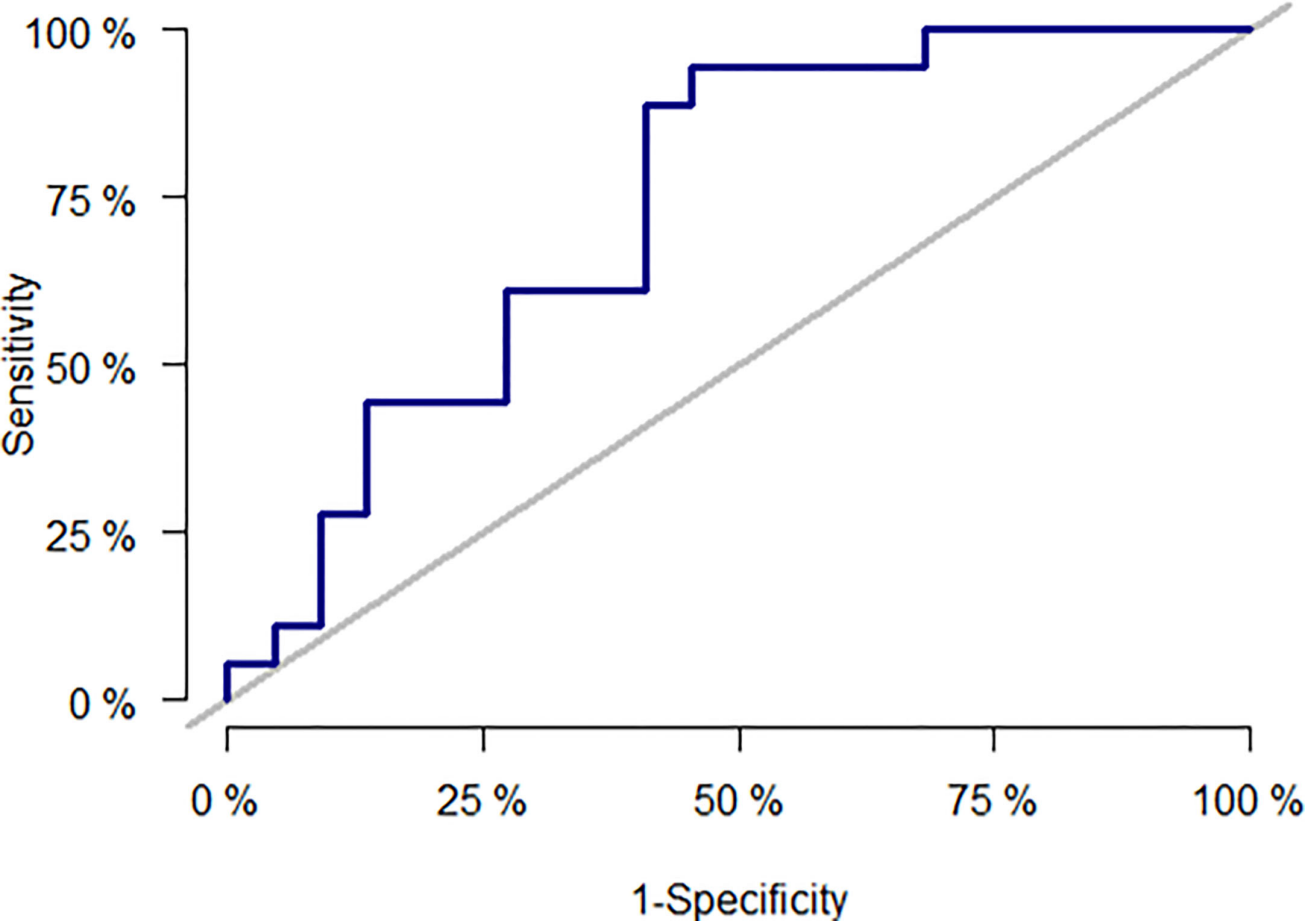
Receiver operating characteristics (ROC) curve for the best performing model consisted of age, type of transplantation, CMV serostatus, and Δ Poly I:C-induced IL-12.

The best performing model consisted of age, type of transplantation, CMV serostatus, and Δ Poly I:C-induced IL-12p40. The discriminatory power of the final prediction model was assessed by a ROC curve with AUC (**Figure 5**). The final prediction model had an average AUC of 77% (61–92) compared to a prediction model including only age, type of transplantation, and CMV serostatus yielding an average AUC of 70% (52–88) (**Table 5**).

**Figure 6:**
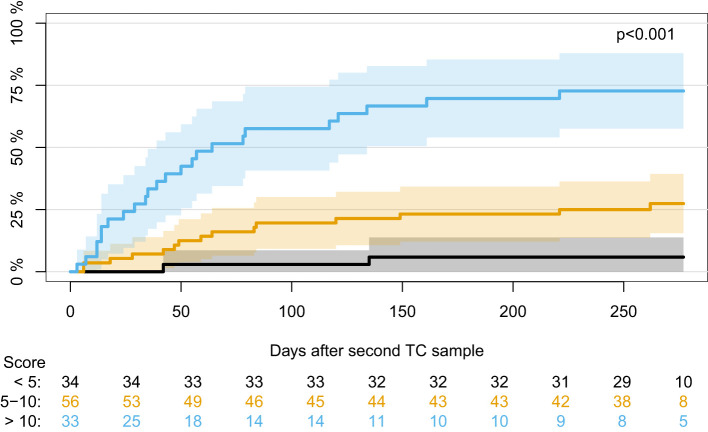
Cumulative incidence of first positive herpes virus PCR test during the first year post-transplantation in participants divided according to risk score using cut-off values of 5 and 10.

Using the covariates from the best performing model (age, type of transplantation, CMV serostatus, and Δ Poly I:C-induced IL-12p40), a model for predicting positive herpes virus PCR tests post-transplantation was made using the entire dataset. From this model, risk scores were extracted, and the participants were divided into three risk groups according to risk score using cut-off values of 5 and 10. Participants with a risk score <5 (28% of the cohort), 5-10 (45% of the cohort), and >10 (27% of the cohort) had a cumulative incidence of having a positive herpes virus PCR test at 6%, 25%, and 73%, respectively (p<0.001) (**Figure 6**). PPV and NPV for risk scores >10 were 0.81 and 0.73, respectively. PPV and NPV for a model including only age, type of transplantation, and CMV serostatus compared to a model including these covariates together with Δ Poly I:C-induced IL-12p40 can be seen in the supplementary material (**Supplementary Table S5**).

In a sensitivity analysis, including only participants with CMV serotype R+, the model including Poly I:C-induced IL-12p40 at three months post-transplantation performed best (**Supplementary Table S6**).

To further investigate the best performing model (age, type of transplantation, CMV serostatus, and Δ Poly I:C-induced IL-12p40), a sensitivity analysis was performed in the 81 (66%) participants with both leucocyte and lymphocyte count at three months post-transplantation were either leucocyte or lymphocyte counts was added to the model to assess if this increased the predictive ability of the model. The model, including age, type of transplantation, CMV serostatus, Δ Poly I:C-induced IL-12p40, and leucocyte counts, performed best and yielded an average AUC of 81% (63–98) (**Supplementary Table S7**).

### 3.6 A subanalysis of only positive cytomegalovirus PCR test

Among the 41 participants with a positive PCR test between three months and 12 months post-transplantation, 36 participants (88%) had a positive CMV PCR test. In a sensitivity analysis investigating only positive CMV PCR tests, these 36 participants had increased cytokine concentrations of several cytokines at three months post-transplantation compared to participants without subsequent positive CMV PCR tests prior to the correction for multiple comparisons (**Table 6**). However, after correction, none of the differences were significant. Furthermore, sensitivity analyses were conducted in participants with CMV serostatus R+ and D+/R- (**Table 6**).

**Table 6:** Immune function in participants with and without positive CMV PCR tests.

**All p-values are prior to Benjamini-Hochberg. No p-values were significant after correction**.			
	All participants (n = 123)	Participants with CMV serotype R+ (n = 86)	Participants with CMV serotype D+/R- (n = 23)
Cytokine concentration three months post-transplantation	↓ R848-induced IFN-γ (p=0.029) ↓ R848-induced IL-12 (p=0.038)	↑ Poly I:C-induced IL-12 (p=0.038) ↑ Poly I:C-induced IFN-α (p=0.009) ↓ R848-induced IL-8 (p=0.031) ↓ R848-induced IFN-γ (p=0.010)	↑ Poly I:C-induced IL-17A (p=0.037)
Changes in cytokine concentration from pre-transplantation to three months post-transplantation	↑ Poly I:C-induced IL-17A (p=0.026)	↑ Poly I:C-induced IL-12 (p=0.037) ↑ Unstimulated IL-8 (p=0.025) ↑ Unstimulated IFN-γ (p=0.041)	–

↓/↑: Lower/higher induced cytokine concentrations in participants with subsequent positive CMV PCR tests compared to participants without subsequent positive CMV PCR tests.

Likewise, when comparing the changes in cytokine concentration from pre-transplantation to three months post-transplantation, four induced cytokine concentrations differed between participants with or without later positive CMV PCR test prior to correction for multiple comparisons (**Table 6**). However, there were no significant differences after correction.

Furthermore, a sensitivity analysis with a prediction model for positive CMV PCR tests was conducted. Six cytokines were significantly associated with subsequent positive CMV PCR tests when adjusted for age, type of transplantation, and CMV serostatus: High absolute concentrations at three months post-transplantation of Poly I:C-induced IFN-α, IL-12p40, and IL-10 and increased positive changes (Δ) in Poly I:C-induced IFN-α and IL-12p40, and R8484-induced IL-1β. The models were tested against each other, and the prediction model yielding the best average Brier score was the model including Δ Poly I:C-induced IL-12p40 adjusted for age, type of transplantation, and CMV serostatus (**Supplementary Table S8**). The prediction model had an average AUC of 77% (61–92). Risk scores were extracted from the model, and participants were divided into three risk groups. Participants with a risk score <5 (32% of the cohort), 5-10 (36% of the cohort), and >10 (32% of the cohort) had a cumulative incidence of having a positive CMV PCR test of 0%, 25%, and 62%, respectively (p<0.001).

## 4 Discussion

In this prospective study, we found a high incidence of positive herpes virus PCR tests in a cohort of liver, kidney, and lung transplant recipients. Over one third of the recipients had a positive herpes virus PCR test within the first year post-transplantation. Herpes virus reactivation is closely linked to immune function, but we found no major immunological differences between recipients with and without later positive herpes virus PCR tests using a functional immunoassay. However, when combining the assessment of the immune function with traditional risk factors, we made a prediction model for positive herpes virus PCR tests with an acceptable AUC.

We found a high incidence of positive herpes virus PCR tests despite the use of antiviral prophylaxis, with CMV being the main contributor. This finding was in line with previous studies showing a high incidence of late-onset CMV after the discontinuation of antiviral prophylaxis (1, 12, 25). At our transplantation center, both antiviral prophylaxis and preemptive therapy are used to prevent herpes virus infections [termed “surveillance after prophylaxis” (1)]. In accordance with our finding, CMV viremia was found in 30.6% of kidney transplant recipients in a cohort also using surveillance after prophylaxis (26). Additionally, we found a low incidence of HSV and VZV comparable to recipients receiving prophylaxis in a previous large study of 2781 SOT recipients and a meta-analysis of VZV incidence (27, 28). Contrary to our findings, previous studies systematically screening for EBV found high incidences of EBV viremia ranging from 33.9-56% in the first year post-transplantation (29–31). However, these studies screened both EBV IgG-positive and IgG-negative recipients, which may have caused the discrepancy since only EBV IgG-negative recipients are screened for EBV at our center, meaning that subclinical latent infection will not be registered.

We assessed immune function three months post-transplantation and the change from pre-transplantation to post-transplantation, using a functional immunoassay, and found no difference in immune function in recipients with and without later positive herpes virus PCR tests. This finding contrasts a previous study reporting decreased cytokine concentration for several cytokines after TLR stimulation in recipients later developing CMV (32). However, the study was conducted in a cohort of 24 CMV D+/R- recipients. D+/R- serostatus is associated with a high risk of donor-derived CMV, which may, in part, have caused the discrepancy.

Dysfunction in innate and adaptive immune responses increased the risk of herpes virus infection in previous studies (1, 33–36). We found associations between five cytokines and later positive herpes virus PCR tests in our adjusted models. All five associated cytokines were after stimulation with either ligand for TLR-3 (Poly I:C) or TLR-7/TLR-8 (R848). TLR-3 and TLR-7/TLR-8 are endosomal pattern-recognizing receptors (PRR) reacting on double-stranded and single-stranded RNA, respectively (37). Despite herpes viruses being DNA viruses, CMV, EBV, HSV, and VZV are all sensed by TLR-3, likely due to the production of double-stranded RNA during transcription, and EBV also by TLR-7/TLR-8 (38). Furthermore, previous studies have found associations between TLR polymorphisms, including TLR-3, and CMV infection, highlighting the importance of TLR signaling in herpes virus infections (39–43).

The immune function was assessed by the TLR-induced production of selected inflammatory and regulatory cytokines. IL-12 is a central player in the Th1 immune response against intracellular pathogens such as viruses (37). IL-12 increases the IFN-γ production in NK cells and T cells, leading to increased macrophage activation and Th1 T cell polarization (37). IL-12 is important in the antiviral response against HSV (44) and is increased in patients with Herpes Zoster (45). Furthermore, a previous study found that endogenous IL-12 is a key factor in driving a CD4+ T cell response against CMV in lung transplant recipients, and that impaired T cell proliferation in CMV relapse could be reduced using exogenous IL-12 and IL-2 together with the CMV antigen pp65 (46). Collectively, and in accordance with our findings, there is evidence to support a biological role for IL-12 in immune responses to herpes viruses, and IL-12 would theoretically be a useful marker for the prediction of herpes virus infections.

The risk of herpes virus reactivation in SOT recipients is linked to both the previous herpes-specific immunity, risk factors for infections, and the overall state of immunosuppression (14, 47, 48). Therefore, we made a prediction model using traditional risk factors such as CMV serostatus combined with an assessment of the immune function against intracellular pathogens (increase in IL-12p40 concentration). This model performed well with an AUC of 77%, which according to statistical literature is considered acceptable (49), and exceeded the average performance of a model including only the traditional risk factors.

In our model, increased Poly I:C-induced IL-12p40 concentrations from pre- to post-transplantation were associated with a higher risk for positive herpes virus PCR tests. This association may be caused by uncontrolled inflammation, possibly from herpes virus infection below the detection limit, triggering increased production of IL-12 via positive feedback mechanisms from circulating pro-inflammatory cytokines such as INF-γ (50). Furthermore, a previous study found increased IL-12p70 in kidney transplant recipients with CMV serotype R+ compared to serotype R- one year after CMV infection, suggesting that latent CMV is associated with increased IL-12 levels post-transplantation, which may, in part, explain findings in our cohort where 70% of the participants are CMV serotype R+ (51). However, then CMV serotype R+ participants in our cohort were investigated alone, a similar trend was found with increased IL-12 in participants with subsequent positive herpes virus PCR tests prior to correction for multiple comparisons, suggesting that poor control of latent CMV infection is associated with increased IL-12 production prior to reactivation.

The traditional risk factors used in our model were CMV serostatus, type of transplantation, and recipient’s age at transplantation. Sex was assessed and not included since it did not affect the risk in our cohort. The risk factors were chosen to reflect common risk factors in the literature, where CMV serostatus is a well-established risk factor for CMV (1, 3, 11, 33, 47). Furthermore, differences in the risk of herpes virus infection across different types of transplantation have been reported for CMV and VZV. In CMV infections, lung and small intestine transplant recipients are considered at the highest risk, possibly due to a higher amount of lymphoid tissue and/or donor tissue macrophages, whereas lung and heart transplant recipients are at greater risk of VZV infection or reactivation, presumably linked to heavier immunosuppression (6, 28, 33, 47, 52). Lastly, age was found to be a risk factor for CMV and VZV but not for EBV and HSV (6, 11, 27–30, 47, 53–56).

Other studies additionally found rejection, leucopenia, and certain types of immunosuppressive drugs, such as ATG and MMF, to be risk factors for herpes virus infections (1–3, 5, 6, 9, 11, 27, 29, 30, 33, 53, 54). We found that adding leucocyte count at three months to our prediction model increased the predictive value of the model, highlighting the possibility of implementing these factors in future studies of larger cohorts.

The study’s strengths include an assessment of the immune function using a standardized immune assay in a cohort of SOT recipients. Furthermore, systematic screening of CMV and EBV was used and combined with the nationwide register of PCR tests collected on clinical suspicion of herpes virus infection. This combined method resulted in a precise estimate of the incidence of positive herpes virus PCR tests. However, VZV or HSV infections treated solely based on the symptoms will be missing, and our incidence may, therefore, be conservative. Furthermore, we combined traditional risk factors for herpes virus infection with an assessment of the immune function, making a personalized prediction model with higher accuracy than a model including only the traditional risk factors. A similar personalized prediction model may in the future be used to stratify transplant recipients in risk groups, where high-risk recipients could be screened more intensely than low-risk recipients, thereby possibly decreasing symptomatic herpes virus disease and lowering cost.

The study has limitations. First, the lack of an external validation cohort limits the generalizability of the study. However, we did internal validation when investigating which cytokine to include in the prediction model, thereby reducing bias. Second, the study cohort was heterogeneous. Nevertheless, it was representative of our transplantation center. Third, to gain power, all herpes viruses were grouped together despite biological differences. Fourth, we investigated positive PCR results instead of clinical infection, and we do not have information about the outcome of the infections or the proportion that needed antiviral treatment. However, considering the low incidence of herpes virus infections after the introduction of prophylaxis and preemptive treatment and the close relationship between viral load and CMV disease, the authors of a recent meta-analysis concluded that viral load could be a preferred endpoint in future CMV trials (57). Firth, we did not collect data on the immunosuppression between three and 12 months post-transplantation. Lastly, the study did not have sufficient power to assess prediction in the group of D+/R- participants.

In conclusion, the incidence of positive herpes virus PCR tests was high the first year post-transplantation. We found no differences in the immune function in recipients with later positive herpes virus PCR tests compared to recipients without positive tests. Importantly, combining traditional risk factors for herpes virus infections with an assessment of the immune function post-transplantation allowed for a prediction model for positive herpes virus PCR tests post-transplantation with an acceptable AUC and with better performance than a model based on traditional risk factors alone. Thus, moving towards personalized immunological risk assessment of herpes viruses in SOT recipients in the future.

## Data availability statement

The datasets generated and analyzed during the current study are not publicly available because of ethical and privacy restrictions established by Danish legislation. Requests to access the datasets should be directed to Sdn@dadlnet.dk.

## Ethics statement

The study including human participants was reviewed and approved by the Health Authorities (3-3013-1060/1), the Regional Committee on Health Research Ethics (H-17024315), and the Data Protection Agency (RH-2016-47, RH-2015-04, I-Suite 03605). The study has been registered at clinicaltrials.gov (NCT03847285). The patients/participants provided their written informed consent to participate in this study.

## Author contributions

DM, SS, MP, FG, JAL, JK, SO, AR, and SN participated in the research design. DM, SS, MP, FG, OR, DR, NA, JAL, JL, JK, SO, AR, and SN collected the data. DM and TS did statistical analyses. DM and SN wrote the first draft. All authors contributed to the article and approved the submitted version.
