# Supplementary material for: Prediction of herpes virus infections after solid organ transplantation: a prospective study of immune function
Source: Front Immunol. 2023 Jul 3;14:1183703. doi: 10.3389/fimmu.2023.1183703 (PMC10351284; doi:10.3389/fimmu.2023.1183703)
Supplement: Supplementary file 1 [file DataSheet_1.pdf]

Supplementary Material

Prediction of herpes virus infections after solid organ transplantation: A prospective study of the immune function

Dina Leth Møller, MD, Søren Schwartz Sørensen, MD, DMSc., Omid Rezahosseini, MD, Daniel Bräuner Rasmussen, BSc, Nicoline Stender Arentoft, MD, Josefine Amalie Loft, MD, Michael Perch, MD; Finn Gustafsson, MD, DMSc., Jens Lundgren, MD, DMSc., Thomas Scheike, DSc; Jenny Knudsen, MD, DMSc., Sisse Rye Ostrowski, MD, PhD, DMSc.; Allan Rasmussen, MD; Susanne Dam Nielsen, MD, DMSc.

*** Correspondence:** Corresponding Author at:
Viro-immunology Research Unit, Department of Infectious Diseases 8632, Rigshospitalet, University of Copenhagen, Copenhagen, Denmark.

E-mail address: Sdn@dadlnet.dk, Phone: (+45) 35 45 0859, Fax: (+45) 35456648

Postal Address: Viro-immunology Research Unit, Department of Infectious Diseases 8632, Rigshospitalet, University of Copenhagen, Blegdamsvej 9B, DK-2100 Copenhagen Ø, Denmark.

1 Supplementary Data

1.1 Supplementary Table S1: Reason for transplantation

	All participants<br><br>N = 123	Liver transplanted participants<br><br>N = 67 (54%)	Kidney transplanted participants<br><br>N = 32 (26%)	Lung transplanted participants<br><br>N = 24 (20%)
Reason for transplantation				
Autoimmune liver disease<br><br>Subgroups of autoimmune disease<br>*Primary sclerotic cholangitis*		37 (55%)[a]<br><br><br>29 (43%)[a]	-	-

Primary biliary cholangitis<br>*Autoimmune hepatitis*<br><br>Hepatocellular carcinoma<br>Cirrhosis<br>Metabolic liver disease<br>Hepatitis C<br>Other	6 (9%)[a]<br>7 (10%)[a]<br><br>4 (6%)[a]<br>13 (19%)[a]<br>2 (3%)[a]<br>2 (3%)[a]<br>13 (19%)[a]		
Cystic kidney disease<br>Diabetes<br>Glomerulonephritis<br>Vascular & hypertensive<br>Other and unknown	-	9 (28%)<br>0 (0%)<br>13 (41%)<br>2 (6%)<br>8 (25%)	-
α-1-antitrypsin deficiency<br>Allergic alveolitis and eosinophilic pneumonia<br>Idiopathic pulmonary fibrosis<br>Sarcoidosis<br>Cystic fibrosis<br>Other	-	-	5 (21%)<br>4 (17%)<br><br>5 (21%)<br>3 (12%)<br>1 (4%)<br>6 (25%)

1.2 Supplementary Table S2: Brier and AUC scores, including IFN-α

Model	Average Brier Score (lower-upper score)	Average AUC Score (lower-upper score)
Age, type of transplantation, and CMV serostatus at transplantation	23.8 (14.9-32.7)	62.6 (39.8-85.3)
+ Poly I:C-induced IFN-α at three months	23.9 (14.4-33.3)	62.8 (40.7-84.8)
+ Poly I:C-induced IL-12 at three months	22.4 (13.9-31.0)	66.9 (45.7-87.9)
+ R848-induced IL-17A at three months	23.4 (14.5-32.1)	64.2 (42.2-86.2)
+ Change in Poly I:C-induced IFN-α	22.9 (13.8-32.0)	65.7 (43.8-87.5)
+ Change in Poly I:C-induced IL-12	23.2 (14.5-32.0)	65.2 (43.6-86.8)

+ Change in R848-induced IL-17A	26.0 (16.3-35.8)	60.4 (37.6-83.2)
+ Change in R848-induced IL-1β	25.4 (15.1-35.6)	62.5 (40.0-85.0)
+ Change in R848-induced TNF-α	24.6 (14.8-34.4)	64.6 (42.5-86.6)

1.3 Supplementary Table S3: Brier and AUC scores without the five participants with concurrent herpes virus infection at time of blood sampling

Model	Average Brier Score (lower-upper score)	Average AUC Score (lower-upper score)
Age, type of transplantation, and CMV serostatus at transplantation	19.2 (11.7-26.41)	72.7 (55.7-89.8)
+ Poly I:C-induced IL-12 at three months	18.7 (11.0-27.0)	73.9 (56.9-90.8)
+ Poly I:C-induced IL-10 at three months	19.3 (11.6-26.8)	71.9 (54.7-89.2)
+ R848-induced IL-17A at three months	19.1 (11.3-25.9)	73.2 (56.0-90.3)
+ Change in Poly I:C-induced IL-12	18.4 (11.0-26.5)	74.8 (57.9-91.6)
+ Change in R848-induced IL-17A	18.7 (10.9-26.6)	74.0 (56.9-91.2)
+ Change in R848-induced IL-1β	18.9 (11.1-26.5)	73.9 (56.8-90.9)
+ Change in R848-induced TNF-α	18.5 (10.5-28.2)	75.5 (59.0-92.0)
+ Change in R848-induced IL-6	19.9 (11.6-27.3)	74.3 (58.2-90.5)
+ Change in LPS-induced IL-17A	19.2 (11.3-27.3)	72.0 (54.3-89.8)

1.4 Supplementary Table S4: Brier and AUC scores without the three participants with one year of antiviral chemoprophylaxis

Model	Average Brier Score (lower-upper score)	Average AUC Score (lower-upper score)
Age, type of transplantation, and CMV serostatus at transplantation	19.3 (12.1-26.9)	72.3 (52.5-86.6)
+ Poly I:C-induced IL-12 at three months	18.6 (11.3-26.6)	73.9 (54.6-87.9)
+ Change in Poly I:C-induced IL-12	18.4 (11.1-25.9)	74.7 (55.5-88.3)
+ Change in R848-induced IL-17A	20.8 (12.7-25.7)	70.8 (50.9-85.1)
+ Change in R848-induced IL-1β	20.6 (12.4-28.9)	70.7 (50.7-85.2)
+ Change in R848-induced TNF-α	19.9 (12.0-28.7)	73.1 (53.8-86.9)
+ Change in R848-induced IL-6	19.7 (12.1-27.8)	72.3 (52.6-85.9)

1.5 Supplementary Table S5: Sensitivity, specificity, PPV, and NPV

Sensitivity, specificity, positive and negative predictive values								
Cut-off values	Sensitivity and Specificity				Positive and negative predictive values			
	Baseline		+ Δ IL-12		Baseline		+ Δ IL-12	
	Sen	Spe	Sen	Spe	PPV	NPV	PPV	NPV
>2	0.17	1.00	0.18	1.00	1.00	0.38	1.00	0.38
>4	0.24	1.00	0.27	0.95	1.00	0.40	0.92	0.39
>5	***0.34***	***0.95***	***0.39***	***0.95***	***0.93***	***0.42***	***0.94***	***0.44***
>6	0.54	0.80	0.56	0.93	0.85	0.46	0.94	0.51
>7	0.62	0.73	0.68	0.73	0.82	0.49	0.84	0.54
>8	0.78	0.56	0.79	0.63	0.78	0.56	0.81	0.60

>10	***0.89***	***0.49***	***0.89***	***0.59***	***0.78***	***0.69***	***0.81***	***0.73***
>20	0.95	0.22	0.95	0.29	0.71	0.69	0.73	0.75
>30	1.00	0.05	0.99	0.10	0.68	1.00	0.69	0.80
>40	-	-	1.0	0.05	-	-	0.68	1.00

1.6 Supplementary Table S6: Brier and AUC scores for models, only including CMV serotype R+ participants (n=86)

Model	Average Brier Score (lower-upper score)	Average AUC Score (lower-upper score)
Age, type of transplantation, and CMV serostatus at transplantation	22.8 (13.8-31.9)	54.2 (28.1-80.4)
+ Poly I:C-induced IL-12 at three months	20.0 (11.1-28.9)	62.8 (38.6-87.0)
+ Poly I:C-induced IL-10 at three months	23.2 (13.7-32.7)	54.8 (30.0-79.7)
+ Change in Poly I:C-induced IL-12	20.2 (11.7-28.7)	64.8 (42.3-86.9)
+ Change in R848-induced IL-1β	23.6 (13.7-33.5)	51.2 (24.3-78.2)

1.7 Supplementary Table S7: Brier and AUC scores for models, including leucocyte or lymphocyte counts (n=81)

Model	Average Brier Score (lower-upper score)	Average AUC Score (lower-upper score)
Age, type of transplantation, and CMV serostatus at transplantation	21.3 (12.4-30.2)	68.7 (45.6-91.8)
+ Change in Poly I:C-induced IL-12	19.5 (10.9-28.0)	75.5 (55.0-95.8)

+ Change in Poly I:C-induced IL-12 **and leucocyte count**	16.7 (8.5-24.9)	81.2 (63.4-98.1)
+ Change in Poly I:C-induced IL-12 **and lymphocyte count**	19.9 (10.7-29.1)	74.2 (53.5-94.9)

1.8 Supplementary Table S8: Brier and AUC scores for positive CMV PCR tests

Model	Average Brier Score (lower-upper score)	Average AUC Score (lower-upper score)
Age, type of transplantation, and CMV serostatus at transplantation	20.0 (12.0-27.9)	71.3 (53.8-88.7)
+ Poly I:C-induced IL-12 at three months	19.2 (11.3-27.1)	74.5 (57.7-91.3)
+ Poly I:C-induced IL-10 at three months	19.6 (11.7-27.6)	72.3 (55.3-89.3)
+ Change in Poly I:C-induced IL-12	18.7 (10.8-26.5)	76.7 (60.94-92.4)
+ Change in R848-induced IL-1β	20.2 (11.6-28.7)	69.8 (51.5-88.2)

2 Supplementary Figures

2.1 Supplementary Figure S1

Cox proportional hazard model

$$h_i(t) = h_0(t) * \exp\left(\beta_1 * x_{i1} + \beta_2 * x_{i2} + \cdots + \beta_n * x_{in}\right)$$

Final prediction model

$$h_i(t) = h_0(t) * \exp\left(age\ at\ tx * 0.008 + \begin{pmatrix} Liver\ tx * 0.175 \\ Kidney\ tx * 0 \\ Lung\ tx * 0.572 \end{pmatrix} + \begin{pmatrix} D(pos)/R(pos) * 0 \\ D(pos)/R(neg) * 1.0180 \\ D(neg)/R(pos) * -0.106 \\ D(neg)/R(neg) * -18.559 \end{pmatrix} + \Delta\, IL - 12 * 0.003 \right)$$

Prediction score from the linear predictions

$$\text{Prediction score} = \exp\left(age\ at\ tx * 0.008 + \begin{pmatrix} Liver\ tx * 0.175 \\ Kidney\ tx * 0 \\ Lung\ tx * 0.572 \end{pmatrix} + \begin{pmatrix} D(pos)/R(pos) * 0 \\ D(pos)/R(neg) * 1.0180 \\ D(neg)/R(pos) * -0.106 \\ D(neg)/R(neg) * -18.559 \end{pmatrix} + \Delta\, IL - 12 * 0.003 \right)$$

Supplementary Figure S1: The formula for the final prediction score for positive herpes virus PCR tests.

2.2 Supplementary Figure S2

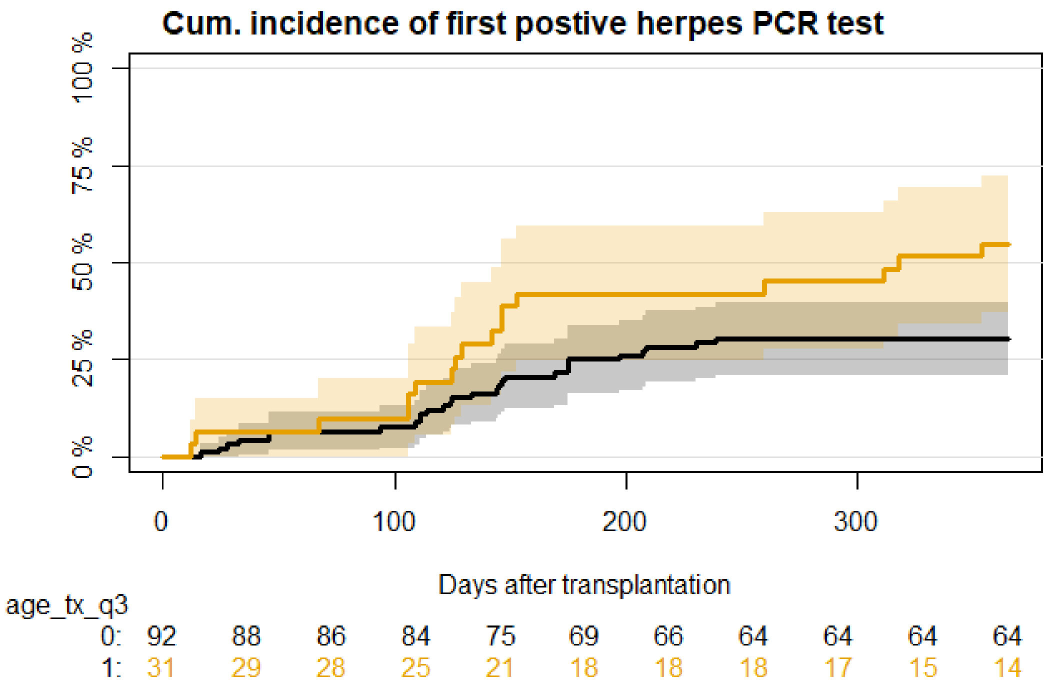

Supplementary Figure S2: Cumulative incidence of first positive herpes virus PCR test during the first year post-transplantation stratified on age above/below the 3rd quantile.

2.3 Supplementary Figure S3

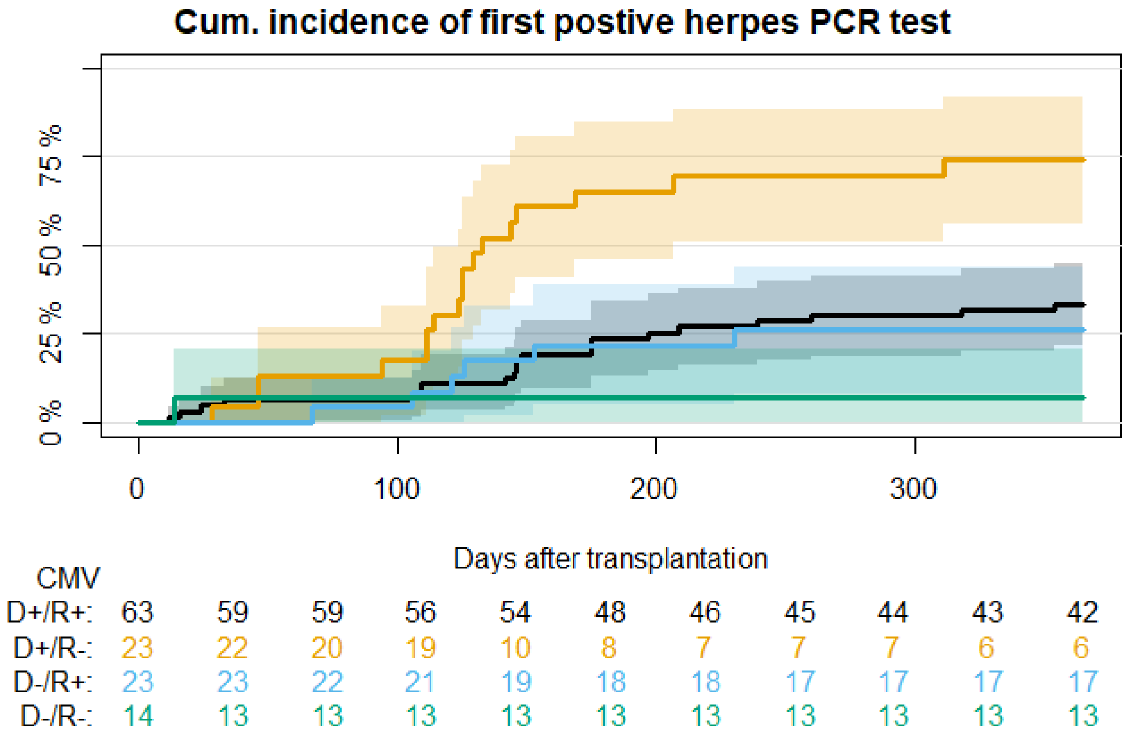

Supplementary Figure S3: Cumulative incidence of first positive herpes virus PCR test during the first year post-transplantation stratified by CMV serostatus.

2.4 Supplementary Material Figure S4

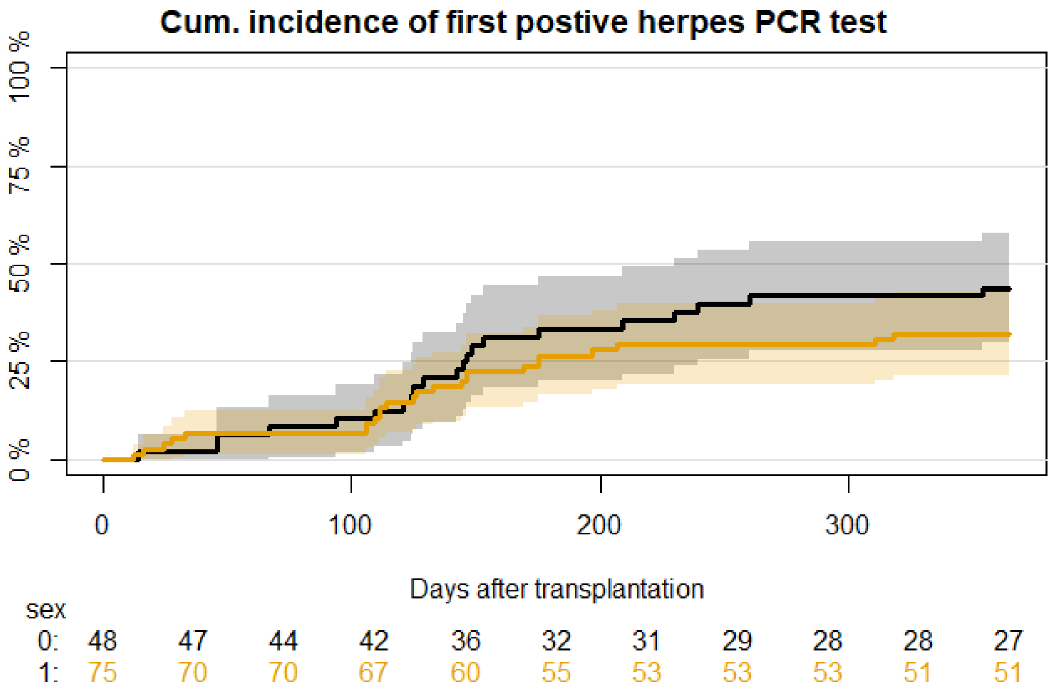

Supplementary Material Figure S4: Cumulative incidence of first positive herpes virus PCR test during the first year post-transplantation stratified by sex.

2.5 Supplementary Material Figure S5

A)

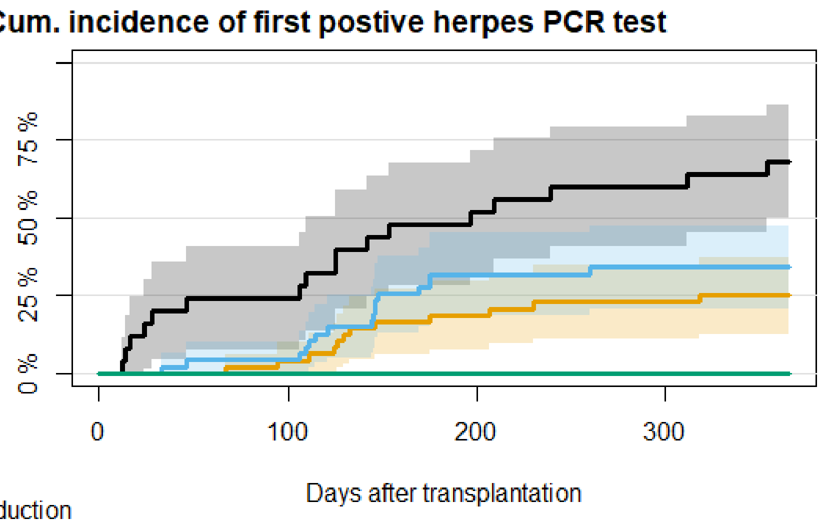

Induction											
ATG:	25	20	19	17	14	13	11	10	10	9	8
Basiliximab:	48	48	47	46	41	39	38	37	37	36	36
No induction therapy:	47	46	45	43	38	32	32	32	31	31	31
Other:	3	3	3	3	3	3	3	3	3	3	3

B)

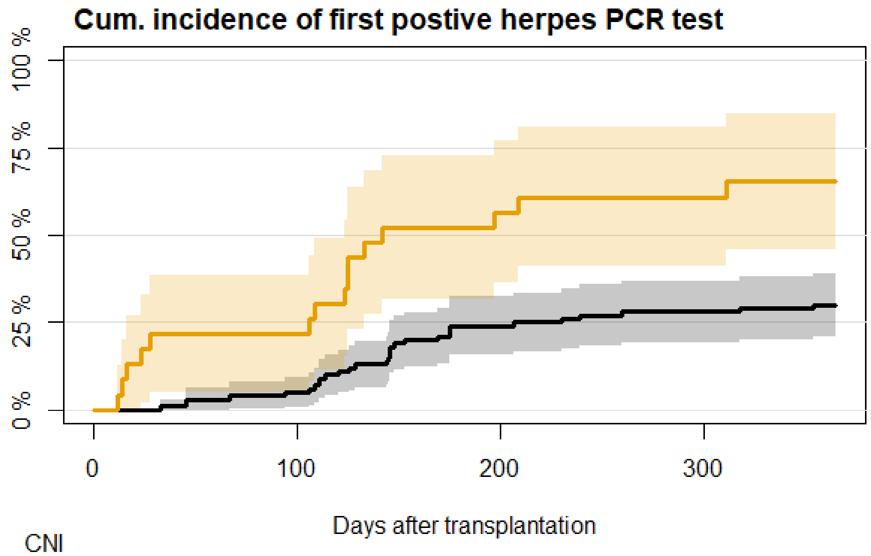

CNI											
Tacrolimus:	100	99	96	93	85	76	75	73	72	71	70
Ciclosporin:	23	18	18	16	11	11	9	9	9	8	8

C)

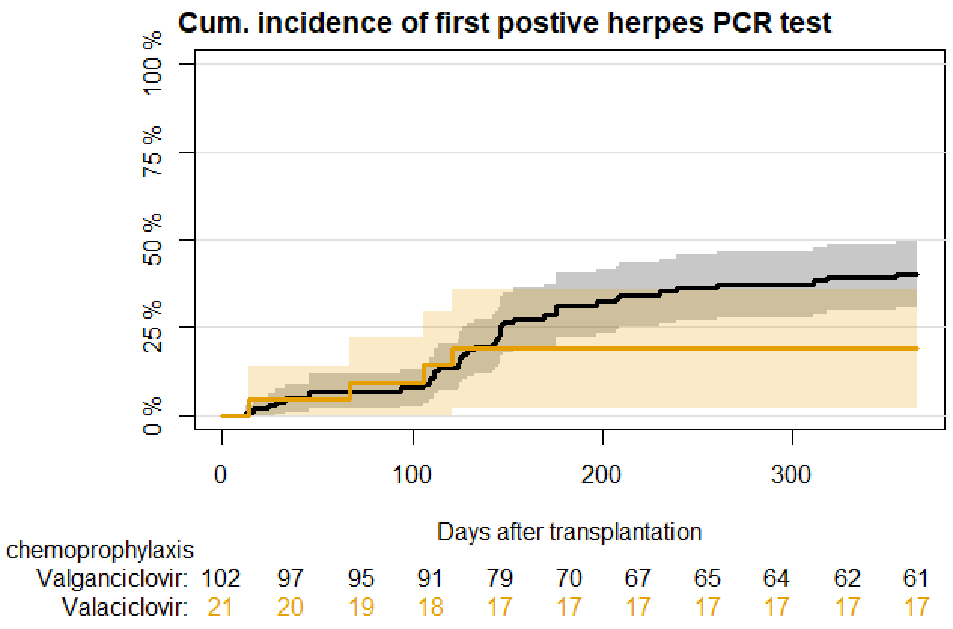

Supplementary Material Figure S5: Cumulative incidence of first positive herpes virus PCR test during the first year post-transplantation stratified by A) the type of induction therapy used, B) the type of calcineurin inhibitor used during the first three months, or C) the type of antiviral chemoprophylaxis used.
